# A Review of Recent Studies on the Antioxidant Activities of a Third-Millennium Food: *Amaranthus* spp.

**DOI:** 10.3390/antiox9121236

**Published:** 2020-12-05

**Authors:** Seon-Joo Park, Anshul Sharma, Hae-Jeung Lee

**Affiliations:** 1 Department of Food and Nutrition, College of Bionanotechnology, Gachon University, Gyeonggi-do 13120, Korea; chris0825@gachon.ac.kr (S.-J.P.); anshulsharma@gachon.ac.kr (A.S.); 2 Institute for Aging and Clinical Nutrition Research, Gachon University, Gyeonggi-do 13120, Korea

**Keywords:** amaranth, antioxidant, hydrolysate, bioactive peptides, radical scavenging

## Abstract

Amaranth (*Amaranthus* spp.) plant commonly refers to the sustainable food crop for the 21st century. The crop has witnessed significant attention in recent years due to its high nutritional value and agronomic advantages. It is a relatively well-balanced cosmopolitan food that is a protector against chronic diseases. Usually, the antioxidant activities of amaranth are held responsible for its defensive behavior. Antioxidant activity of plants, generally, is attributed to their phytochemical compounds. The current interest, however, lies in hydrolysates and bioactive peptides because of their numerous biological functions, including antioxidant effect. While the importance of bioactive peptides has been progressively recognized, an integrated review of recent studies on the antioxidant ability of amaranth species, especially their hydrolysates and peptides has not been generated. Hence, in this review, we summarize studies focused on the antioxidant capacity of amaranth renewal over the period 2015–2020. It starts with a background and overall image of the amaranth-related published reviews. The current research focusing on in vitro, in vivo, and chemical assays-based antioxidant activity of different amaranth species are addressed. Finally, the last segment includes the latest studies concerning free radical scavenging activity and metal chelation capacity of amaranth protein hydrolysates and bioactive peptides.

## 1. Introduction

The perceived deficiencies of essential vitamins and minerals present a significant restriction on human health and economic growth [1]. Considering the importance of plants as one of the leading suppliers of bioactive dietary compounds, recent research focusing on superfoods as a nutraceutical and natural defender against chronic disorders has gained considerable attention.

Currently, there is a great interest in *Amaranthus*, a third-millennium tropical food plant. The genus *Amaranthus* (L.) belongs to the family Amaranthaceae, order Caryophyllales, and includes dicotyledonous annual plants and consists of approximately 70 species, which can be classified into grain and vegetable amaranths [2]. Most of the amaranth species are native to America and only 15 species originate from Asia, Africa, Australia, and Europe [3]. The plant is adapted to grow under different agro-climatic conditions and reported to be heat, drought, and pest tolerant [4]. *Amaranthus* spp. can be found in subtropical, tropical, and temperate climate zones around the world [3,5]. The crop demonstrated its capacity to cultivate both as a grain and leafy in those areas and seasons where other crops are unable to flourish [6,7,8].

Amaranth continues to be listed as an ignored and underused crop [9] despite the high nutrient profile of its grain. Amaranth grain consists of proteins (13–22%), lipids (5–13%), dietary fiber (9–14%), vitamins (ascorbic acid, riboflavin, niacin), minerals, and other phytoconstituents including betalains [10,11,12]. Furthermore, the amaranth proteins have a remarkable amount of lysine and methionine relative to typical cereals [13] and advocated to be a substitute natural source of squalene (polyunsaturated triterpene compound). Amaranth grain is gluten-free, making it an ideal food crop for millions of people worldwide. This nutritional value of amaranth is well-balanced and very similar to that recommended by the Food and Agriculture Organization of the United Nations and World Health Organization [14].

Among culinary uses, amaranth can be used as a vegetable and its grain as cereal in several food products such as whole-meal amaranth flour, bread, cookies, pastries, pancakes, cereal flakes, pasta, tortillas, popping, and candies [5,15,16]. Amaranth grain can be fermented, malted to produce beer, and sprouted for salad preparation, whereas its green can be used in salads, meat and fish dishes [17]. Amaranth flour is used in soups, gravies, and stews as a thickener [18]. In addition, amaranth has tremendous potential in the processing of gluten-free products rich in nutrients such as bread, pasta, and confectionery [19]. Nonfood applications of amaranth include laundry starch, cosmetics, paper coatings, and biodegradable films [18].

In ancient times in Mexico, amaranth was regarded as a basic ingredient in the diet of the Mayan and Aztec empires and was used in drinks, therapeutic treatments and combined with maize to make tortillas [20]. In Peru, popped *A. caudatus* (kiwicha) is used to make snack bars known as turrones, and toasted seeds flour is utilized in porridges, soups, and cookies [3,21]. Ethiopians use amaranth to prepare kita (unleavened bread), borde (alcoholic drink), and atmit, a thin porridge for new mothers and babies [22]. In Vietnam, *A. tricolor* leaves, petioles, and young tips have been used by people for potherbs (boiled greens), soup, and salad preparation [23]. Indian food considers amaranth grain a gastronomic substitute to wheat with higher amounts of fiber and protein [24]. Seeds are consumed with boiled rice, used to make “laddoos” and popped grains are used to create a confectionery product after mixing with molasses and honey [25]. The “Breakfast Amaranth” is the most commercial commodity in the United States [5].

One cup (246 g) of cooked amaranth contains 9 g of protein and 5 g of fiber. It also supplies a good amount of minerals, including iron, phosphorus, magnesium, and manganese [26]. Children, high-geared athletes, gluten and lactose intolerant people, diabetic individuals, and coeliacs are potential buyers of amaranth products concerning nutritional aspects [27]. Recently, a randomized control trial evaluated the effect of amaranth processed bread (amaranth 70% and chickpea 30%) and maize bread (roasted and fermented) on 2–5 years old Southern Ethiopian children with anemia for 6 months. Processed amaranth bread showed favorable effects on hemoglobin concentration and can reduce the incidence of anemia [28]. The relatively high price of amaranth grain (USD 0.9–1.0 per kg), which is up to five times and 10 times higher than wheat and maize, respectively, is the aspect that has improved the performance of amaranth so far [29]. Haplessly, this high price received by farmers has also been a major factor in limiting the use of grain to “health” food products, especially in developing countries.

To date, many synthetic antioxidants with high antioxidant properties have been used; however, current trends of consumers have been shifted on ingesting foods containing health-promoting constituents originating from mother nature and without any synthetic additives [30]. Concerning this context, antioxidants rich plants, especially underutilized species, their protein hydrolysates and bioactive peptides with antioxidant properties can be widely utilized [20,31]. The published data on in vitro and in vivo findings revealed that the genus *Amaranthus* has health-protective and therapeutic properties ascribed mainly to its potent antioxidant potential [2,32]. Notably, *Amaranthus caudatus* has been positioned in the top five vegetable plants with antioxidant potential [3]. The excellent antioxidant capacity of amaranth can be owing to the presence of phenolic acids, flavonoids, phytosterols, and squalene. Apart from that, hydrolysates or bioactive peptides from amaranth have gained interest as antioxidant peptides in recent years [33]. Among others, antidiabetic, antihypertensive, immunomodulation, antitumor, and antimicrobial activities of *Amaranthus* spp. bioactive peptides have been reported [3].

Given the growing interest in amaranth, many excellent reviews have been published describing amaranth, a crop of the 21st century. For instance, Venskutonis and Kraujalis (2013), from a comprehensive perspective, discussed composition, antioxidant properties, uses, and processing. Authors highlighted the importance of macro- and micro-constituents of amaranth, its antioxidant capacity, and effects of processing on antioxidant properties up to 2013 [34]. Rastogi and Shukla reviewed the origin, species, botanical description, chemical composition, ethnobotanical uses, breeding approaches, and biological effects [5]. Similarly, Peter and Gandhi reviewed the bioavailability and therapeutic potential of 13 species of amaranth [2]. The antioxidant ability of four *Amaranthus* spp. (*A. spinosus*, *A. viridis*, *A. graecizans*, and *A. hybridus*) was the focus of Adegbola et al. [32] and mainly reviewed in literature before 2015. Coelho et al. [35] evaluated the nutritional and functional attributes and amaranth as microencapsulation material. The review also summarized how digestibility, bioavailability, and bioaccessibility of amaranth could be improved.

On the other hand, literature concerning antioxidant peptides of amaranth is inconsistent. A review by Orona-Tamayo et al. [20] compiled a small section on amaranth (one of the Latin American food crops) derived bioactive peptides showing their multivariate activities, including antihypertensive, anticancer, immunomodulatory, antimicrobial, antidiabetic, hypocholesterolemic, and antioxidant activity of *A. hypochondriacus*. The authors also emphasized to use genetic engineering approaches for enhancing functional attributes of bioactive peptides. Likewise, another recent review compiled the nutritional profile and different biological activities of bioactive peptides of amaranth [3]. Microorganisms usually produce these peptides in the fermentation process and proteolytic enzymes in the gastrointestinal (GI) tract and those with antioxidant properties have huge potential in the food and health sectors [36]. Hence, this review compiles recently published studies on amaranth hydrolysates and peptides and nearly all studies showcased the use of commercially available hydrolytic enzymes, including alcalase, pancreatin, papain, pepsin, flavourzyme, and neutrase. As mentioned later in this review, in some experiments, a combination of two or more enzymes has been used, while in other studies, sequential proteolysis was performed. Though, the potential effect of bacterial strains on the development of antioxidant peptides has been investigated in several scientific studies [31]. Concerning amaranth, a recent study, for the first time, documented the use of lactic acid bacteria (LAB) for the production of bioactive peptides from amaranth and evaluated cardiovascular protecting effects [37].

Notwithstanding these reviews described above, the information on recent studies on antioxidant capacity of *Amaranthus* spp. is fragmentary. Hence, this review includes literature studies published from 2015 to 2020 and includes the antioxidant activities of about 20 different *Amaranthus* spp. using in vitro and in vivo models in the context of their contribution in antioxidant systems and regulation of signaling mechanisms. In the end, this review pays special attention to recent developments targeting antioxidant activities of amaranth-derived hydrolysates or bioactive peptides.

## 2. Approach

The literature search was performed using PubMed, Google Scholar, and Google to search for publications related to *Amaranthus* spp. The following keywords were used “*Amaranthus*”, “amaranth”, “antioxidant activity”, “free radical scavenging”, “hydrolysate”, “peptides from amaranth”, and “bioactive peptide from amaranth”. The objective of this review was to present the published literature (2015–2020) on the antioxidant potential of *Amaranthus* spp.

## 3. Antioxidant Potential of *Amaranthus* spp.

Antioxidants play an essential role in food and pharmaceutical products because of their defensive roles against reactive oxygen species (ROS) produced during metabolic reactions in all living organisms. ROS have been identified as the cause of oxidative stress-mediated chronic diseases. Numerous recent studies have reported the antioxidant activity of *Amaranthus* spp. extracts made from different parts of the plant. The antioxidant activity based on chemical assays is described in Table 1.

### 3.1. Chemical-Based Assays

The methanolic crude extracts of *A. spinosus* leaves were shown to scavenge 2,2-diphenyl-1-picrylhydrazyl (DPPH) radicals and to inhibit linoleic acid oxidation. The authors also reported total phenolic and total flavonoids activity [38]. Antioxidant activity of organic amaranth flour was evaluated using its free and bound form against DPPH radicals; the antioxidant activity of the bound form was found to be much higher compared to the free form. The bound and free antioxidant activities ranged from 91.01% to 98.70% and 1.30% to 8.90%, respectively [39]. The methanol and hot water extracted tannin from flowers, seeds, and leaves of *A. caudatus* were shown to scavenge 2,2’-azino-bis (3-ethylbenzothiazoline-6-sulfonic acid) (ABTS), DPPH radicals and have reducing power. In addition, at 1 mg/mL, the reduced antioxidant power assay values ranged from 0.1 to 1.14. At 100 μg/mL, tannin inhibited 41% of the superoxide radical in a human promyelocytic leukemia (HL-60) cell line and 43.4% in the nitric oxide (NO) levels in macrophage (RAW 264.7) cells. Besides, the expression of superoxide dismutase (SOD) was increased in tannin-treated RAW 264.7 cells. The findings suggest that the amaranth tannin has antioxidant activity and could be a plausible antitumor agent [40].

Amaranth leaf, seed, flower, sprout, and stalk extracts of different species viz., *A. cruentus*, *A. caudatus*, *A. hypochondriacus* were shown to possess antioxidant activity. The antioxidant activity of *A. hypochondriacus* was found to be higher in leaves, with ferric reducing antioxidant power (FRAP) and oxygen radical scavenging activity (ORAC) values of 62.2 μmol ascorbic acid equivalent (AAE)/g dry weight (DW) and 451.4 μmol Trolox equivalent (TE)/g DW, respectively. The lowest antioxidant activity for FRAP and ORAC assays, was reported in *A. hypochondriacus* seeds, with values <1.7 μmol AAE/g DW and <51 μmol TE/g DW, respectively. The antioxidant activity in FRAP and ORAC assays showed a strong correlation with the presence of rutin, betacyanins, as well as other phenolics present in amaranth samples [41].

Amornrit and Santiyanont studied the antioxidant capacity of petroleum ether, dichloromethane, and methanol leaf extracts from *A. lividus* and *A. tricolor;* the DPPH and ABTS radical scavenging activity was examined in vitro. The methanolic extract of *A. lividus* and *A. tricolor* had the greatest DPPH (15.2 ± 1.6 mg vitamin C equivalent antioxidant capacity (VCEAC)/g extract) and the ABTS (25 ± 1.6 mg VCEAC/g extract) radical scavenging activity, respectively. In the second-highest category, the methanolic extract of *A. tricolor* in the DPPH (13.9 ± 0.3 mg VCEAC/g extract) assay and dichloromethane and methanol extracts of *A. lividus* in the ABTS assay (20.9 ± 0.7 and 20.9 ± 0.7 mg VCEAC/g extract, respectively), were reported. The petroleum ether extract of *A. tricolor* had the lowest scavenging values of 1.7 ± 0.9 and 2.1 ± 0.9 mg VCEAC/g extract in the DPPH and ABTS assays, respectively [42]. The methanolic crude extracts from green leaves of *A. caudatus*, *A. viridis*, and *A. lividus* were shown to scavenge DPPH, to inhibit lipid peroxidation (LPO), total antioxidant capacity (TAC) and reducing power (RP) assay was also determined. A strong correlation was found between total phenolic content (TPC) and DPPH assay followed by TAC, RP, and LPO assay [43].

The methanolic extracts of stem and seeds of *A. lividus* and *A. hybridus* were shown to scavenge DPPH radicals, using butylated hydroxytoluene as a standard. The IC_50_ values for *A. lividus* and *A. hybridus* were 93 ± 2.4 and 28 ± 1.8 μg/mL, respectively [44].

Hejazi and Orsat [45] investigated the effect of the malting process (duration and temperature) on the antioxidant activity of *A. caudatus* grains. The results showed that the germination at 26 °C and 48 h significantly increased the TPC and the radical scavenging activity for DPPH and ABTS assays. In another study, TPC in Cristalino and Taray varieties of *A. caudatus* was 32.9 and 35.0 mg gallic acid equivalents (GAE)/g, respectively. Antioxidant activity in the ABTS assay for the germinated seeds was 151.8 mg TE/g sample for Cristalino and 151.3 mg TE/g sample for Taray, whereas, in the DPPH assay, those values were 178.1 and 180.2 mg TE/g sample, respectively. The FRAP values were found to be 132.8 and 136.4 mg TE/g of sample, respectively, for Cristalino and Taray [46]. The ethanolic extracts of *A. viridis* leaves were shown to inhibit LPO, to scavenge NO production, and to scavenge DPPH radicals. The radical scavenging was concentration-dependent and the highest inhibition (%) was observed at a maximum concentration (500 mg/mL) for DPPH, NO, and antilipid peroxidation, with values of 96.4 ± 0.5, 96.3 ± 0.1, and 97.6 ± 0.04, respectively. The extract showed better ability to quench NO [47]. In another study, the crude methanol extracts of leaf, stem, and seed of *A. viridis* were evaluated by the FRAP, DPPH, ferric thiocyanate (FTC), thiobarbituric acid (TBA) assays, and to scavenge NO production. The inhibition of hydroperoxides was similar, as measured by FTC and TBA assays. For DPPH, FRAP, and NO, the percent inhibition was in the order of leaf > seed > stem. Addition of *A. viridis* at 300 μg/mL augmented the scavenging of DPPH by 70%, 58%, and 39%, FRAP by 85%, 72%, and 48%, and NO by 60%, 48%, and 22%, respectively, in leaves, seeds, and stems [48]. Furthermore, *A. spinosus* seed extracts were shown to scavenge DPPH and hydroxyl radicals, though the scavenging capacity was found to be lower than the standard vitamin C [49].

Akin-Idowu et al. [50] compared the phytochemical constituents and antioxidant potential of five species of amaranth, including *A. caudatus*, *A. cruentus*, *A. hybrid*, *A. hypochondriacus*, and *A. hybridus*. The higher DPPH activities viz., 91.4% and 90.2%, were found in *A. hypochondriacus* and *A. cruentus,* respectively. The most increased and lowest ABTS scavenging activities were observed in *A. caudatus* (169.6 mmol TE/100 g) and *A. hybridus* (201.5 mmol TE/100 g). The TAC capacity ranged from 140.2 mg AAE/100 g of *A. caudatus* to 199.9 mg AAE/100 g of *A. hybridus*. The ferric reducing power and iron chelating activity ranged from 0.14 to 0.19 g/100 g and 57.5–66.7%, respectively. Overall, *A. hybridus* was found to have a higher amount of phytoconstituents and antioxidant capacity among all species. Five-grain amaranth species exhibited a high amount of phytochemicals and better antioxidant activities compared to cereals, including barley, corn, millet oat, rice, and wheat. In another study, 15 amaranth species were evaluated for antioxidant activity using ORAC assay. The phenolics ranged from 3.2 to 5.5 mg GAE/g. The antioxidant activity of amaranth leaves ranged from 38 to 90 mmol TE/g fresh weight (FW). The highest and lowest ORAC values were found in *A. hybr.* and *A. viridis*, respectively. Notably, the antioxidant level of all amaranth species was higher than the spinach lettuce, spider flower, and black nightshade [51]. Antioxidant activities of methanolic extracts of *A. cruentus*, *A. caudatus*, *A. hypochondriacus*, *A. hybridus,* and *A. hypochondriacus* X *hybridus* that grew in Mediterranean regions, were evaluated by the DPPH and ABTS assays; antioxidant capacity was found be best for *A. caudatus* in both assays. A positive correlation between the antioxidant capacity and grain yield and a negative correlation with tocopherol fractions was found. In addition, the antioxidant activity was positively correlated with sitosterol, suggesting the alleged role of this compound in the bioactivity [52]. The radical scavenging activity of *A. caudatus* grain sprouts for ORAC assay ranged from 369.5 to 2525.6 mg TE/100 g DW and found to be positively correlated with phonemic compounds. However, germination of *A. caudatus* grain was temperature- and time-dependent, and the temperature had a greater effect on the antioxidant activity than time [53].

The methanolic extracts of *A. spinosus* leaves from different locations (Redeyef, Nasrallah, and Tabarka) were evaluated for scavenging of DPPH and hydrogen peroxide (H_2_O_2_). In applied assays, the samples from Redeyef showed the highest radical scavenging activity [54]. In one of the studies, different extracts (methanol, chloroform, and aqueous) of *A. tricolor* were evaluated for radical scavenging of DPPH and p-nitroso dimethyl aniline (p-NDA) radicals. The methanol extract showed the highest free radical scavenging activity in applied assays; this may be due to the presence of more elevated flavonoids and phenols. The scavenging effect was dose-dependent [55]. In another study, stems of *A. cruentus* were screened for radical scavenging activity against DPPH and ABTS radicals. The study evaluated the antioxidant activity of different stem heights, and plants with 15 cm in height showed the highest radical scavenging activity. The study also suggested using *A. cruentus* extract as a sausage supplement at an optimum dose of 4.87 g for sausage production [56].

Water, ethanol, ethyl acetate, chloroform, *N*-hexane, and *N*-butanol extracts of *A. spinosus* leaves were evaluated for total antioxidant capacity (TAC) and to scavenge DPPH and hydroxyl radicals. The ethyl acetate fraction contained a higher content of total phenols, total flavonoids, and condensed tannin. In addition, the EC_50_ values of ethyl acetate fraction were found to be remarkably lower, with values of 45.5, 27.3, and 30.6 μg/mL for TAC, DPPH, and H_2_O_2_, respectively [57]. Twenty different genotypes (VA1-VA20) of vegetable amaranth (species not defined) were evaluated for radical scavenging of DPPH radicals by Sarker et al. [58]. The highest and lowest antioxidant activity was found in genotype VA3 and VA8 with values of 32.8 TEAC μg/g DW, 14.9 TEAC μg/g DW, respectively. They also found a strong positive correlation among TAC, antioxidant leaf pigments, and foliage yield. The same research group evaluated the impact of soil water stress on phytoconstituents and TAC of selected amaranth genotypes (VA6, VA11, VA14, and VA16), and found substantial changes in phytonutrients and TAC [59]. On a similar line, the effect of drought stress on ROS markers (malondialdehyde (MDA), H_2_O_2_) and physiological parameters in VA6, VA11, VA14, and VA16 was evaluated. Cultivars VA14 and VA16 showed promising results and positive correlations among ROS markers, nonenzymatic antioxidant, and compatible solutes were found [60]. Similarly, the effect of salinity stress (100 mM NaCl, 50 mM NaCl) on phytoconstituents composition and TAC of *A. tricolor* was evaluated by Sarker et al. [61]. The TAC and nutritional quality were remarkably increased following salinity stress, suggesting the significant interrelationships of polyphenols, flavonoids, and vitamins content with the total antioxidant capacity. In two independent studies by similar research groups, polysaccharides were extracted from *A. hybridus* using microwave (AHP-M-1 and AHP-M-2) and hot water (AHP-H-1 and AHP-H-2) extracts and screened for DPPH, hydroxyl, and superoxide anion radicals scavenging, and for iron (III)–iron (II) reduction assay. Total radical scavenging activity per milligram of AHP-M-2 and AHP-H-2 was found to be 6.42 and 6.5, respectively, higher than radical scavenging of vitamin C by 4.5. These findings suggest that polysaccharides from amaranth, specifically *A. hybridus* has vast potential as natural antioxidants [62,63]. Sarker and Oba summarized the nutritional profile and antioxidant capacity of three genotypes of weedy amaranth species. *A. spinosus* genotypes (WAV4, 7, and 9) showed higher scavenging of DPPH and ABTS radicals compared to *A. viridis* genotypes (WAS11, 13, and 15). The highest DPPH and ABTS radical scavenging were observed in genotype WAS13 with values 27.6 and 52.4 TEAC μg/g DW, respectively. On the other hand, the lowest scavenging values 21.9, 48.2 TEAC μg/g DW were recorded in genotype WAV7, for DPPH and ABTS radicals, respectively. A significant positive correlation was found among vitamin C, β-carotene, TPC, total flavonoids content (TFC), DPPH, and ABTS [64].

Researchers and consumers have recently shown interest in the red-colored vegetables for natural antioxidants. Concerning red amaranth, a recent study screened 25 red morphs amaranth species (RA1 to RA25) and their antioxidant activity was evaluated by DPPH and ABTS assays. Among all, genotype RA3, RA5, RA15, RA18, and RA25 showed higher antioxidant activity. The study also found a significant positive association of DPPH, ABTS, TPC, and TFC, among each other, vitamins, and all leaf pigments present in red amaranth [65]. In another study, the same group summarized that the antioxidant capacity of red color amaranth (*A. tricolor*) genotypes was found to be excellent compared to green color (*A. lividus*) genotype, the scavenging values for DPPH and ABTS radicals were 43.8 and 66.6 TEAC μg/g DW, respectively, for red color genotype compared to green color genotype [66]. In continuing research, among 17 genotypes of *A. lividus*, DS40, DS30, and DS26 showed higher scavenging of DPPH and ABTS radicals with values of 26.6, 26.5, 25.2 TEAC μg/g DW and 51.7, 49.6, 45.2 TEAC μg/g DW, respectively [67]. Likewise, GRA4 among 12 genotypes of green morph amaranths leafy vegetable showed highest DPPH and ABTS scavenging activity with values 27 and 48 TEAC μg/g DW, respectively [68]. Another similar research evaluated that among 16 accessions of *A. blitum*, DS3, DS6, DS8, and DS12 displayed the highest antioxidant activity.

The scavenging values for DPPH radicals were 28.6 for DS3 and 29.5 for DS6, DS8, and DS12 μg/g DW and for ABTS radicals the values were around 55 μg/g DW [69]. Overall, extensive in vivo and clinical studies are prerequisites to ascertain the full health-promoting potential of this wonder plant. Amaranth cookies prepared using germinated and raw flour were shown to have antioxidant activity. Chauhan et al. [70] have demonstrated that cookies made from germinated amaranth flour showed the highest scavenging of DPPH radicals compared to raw amaranth flour cookies. Kumar et al. [71] evaluated the antioxidant capacity of coarse seed coat rich fraction of amaranth grain in TAC, DPPH, and ABTS assays. Interestingly, the polyphenolic composition of the crude seed rich fraction was found to be lower while its antioxidant activity was highest. The authors described that the most increased antioxidant activity could be due to the presence of nonphenolic phytoconstituents.

Recently, methanolic extracts of *A. spinosus*, *A. dubius*, *A. viridis*, and *A. tricolor* were evaluated to scavenge DPPH, H_2_O_2_ radicals, and ferric reducing capacity assay. The DPPH radical scavenging activity was found to be higher for *A. spinosus* with an IC_50_ of 63.94 ± 3.72 µg/mL. On the other hand, *A. dubius* showed higher values for H_2_O_2_ scavenging (26.02 ± 1.50 µg/mL) and ferric reducing antioxidant capacity (20.44 ± 0.94 µg/mL). The study also observed higher levels of phenolic contents in *A. spinosus* and high flavonoid content in *A. dubius,* corroborating to their high antioxidant potential. Among all, *A. spinosus* and *A. dubius* also showed better anti-inflammatory and antiproliferative activities [72]. It is well-known that phytoconstituents type and concentration may be influenced by geographical, environmental, and genetic factors, as well as other physiological differences [73]. The quality of extraction is greatly impacted by the polarity of the solution, the length of the extractive process, and the quantitative and qualitative distribution of the compounds. This is why there is no single strategy for knowing an extract’s antioxidant potential, so various techniques with different modes of action need to be used to achieve more systematic and full information. It has been reported that approximately 85% of traditional therapeutic practices involved the use of plant extracts. People are increasingly more interested in the presence of specific compounds with health beneficial properties, thereby giving rise to the production of functionalized food products. Since plant extracts normally exist as a mixture of various forms of bioactive compounds or phytochemicals with different polarities, their isolation remains a major challenge for the bioactive compound detection and characterization process [74]. Extract preparation generally involves the use of organic chemicals, which may have harmful effects in the long run. Thus, new technologies, such as green extraction methods are being developed, where fewer to no organic solvents are used to reduce environmental and health impacts [75]. In addition, most of the plant extract-based studies are published without extract characterization. The characterization of compounds from plant extracts, however, is important to harness its full potential in determining the plausible health benefits of plants and to further contribute to pharmaceutical and biological research. High cost, sophisticated facilities, and technical workforce could be the probable causes.

This paper describes the antioxidant potential of *Amaranthus* spp. and all these studies have confirmed that the high antioxidant activities of amaranth are species-dependent, and thus, on the number of bioactive constituents. The in-depth analysis of health-promoting properties of different extracts of amaranth bioparts has been reviewed elsewhere [2]. Nevertheless, other uses of *Amaranthus* spp. extracts include food additives, cosmetics, antimicrobial, antibiofilm, pharmaceutical, fabrication of nanoparticles, and polymer biocomposites. One of the key causes of skin aging and dermatological disorders is oxidative stress. Several plant extracts used in folk medicine are widely used in the cosmetics field, primarily in antiaging and personal care products [76]. Amaranth’s squalene has a high content of antioxidants and prevents oxidative damage caused by free radicals, especially in the skin [77]. *A. viridis* leaf extract showed antihyaluronidase property [48], this can be utilized as an important antiaging and anti-inflammatory agent. Nanoparticles of silver [78] and iron oxide [79] from amaranth leaf extracts demonstrated antifungal action against plant fungal infections and antibiofilm effects, respectively. Recently, the use of *A. tricolor* leaf extract with polyvinylalcohol has been demonstrated in fabricating a biocomposites polymer as an excellent ultraviolet B-shielding material for aquatic and terrestrial ecosystems [80]. These findings indicate that the different extracts of amaranth bioparts have not only shown the ability for antioxidant capacity but can be utilized in various other applications.

### 3.2. In Vitro Studies

In an in vitro study, partially purified alkaloids (PPA) from *A. viridis* ameliorated the H_2_O_2_-induced oxidative stress in human erythrocytes. The enzymatic and nonenzymatic activities were enhanced in a dose-dependent manner and lipid peroxidation levels were decreased by preventing MDA formation. These findings suggest that alkaloids from *A. viridis* have antioxidant effects (Table 2) [81].

The antioxidant activity of leaf extracts of *A. lividus* and *A. tricolor* has been reported in H_2_O_2_-induced SH-SY5Y (human neuroblastoma) cells. The leaf extracts of both species protected SH-SY5Y cells from ROS stress by downregulating the expression of oxidative stress-related genes such as a receptor for advanced glycation end products, heme oxygenase one, and nuclear factor-kappa-B p65 subunit (Table 2) [42]. Phytoconstituents that activate the nuclear factor erythroid two-related factor two (Nrf2) signaling are being investigated for their potential to become preventive or therapeutic agents for oxidative stress-related diseases. Research has been conducted on evaluating the protecting effect of *A. cruentus* extract against aflatoxin B1 (AFB1) and oxidative stress-induced DNA damage in HepG2 cells. The extracts were shown to inhibit DNA damage in a concentration-dependent manner, and the DNA damage was inhibited by 81% and 57% induced by oxidative stress and AFB1, respectively. Moreover, *A. cruentus* extract was shown to induce antioxidant responsive element (ARE)/Nrf2-mediated antioxidant enzymes expression (Table 2) [82].

### 3.3. In Vivo Studies

In a study, amaranth protein (AI) and cholesterol (Chol) showed protective antioxidant effects in plasma and liver of male Wistar rats. The AI supplementation increased FRAP values by 50%, while 2-thiobarbituric acid (TBA) value was reduced by 70% (plasma) and 38% (liver). A decreased SOD activity in CholAI group plasma by 20% indicates an antioxidant effect due to AI consumption. However, AI supplementation without Chol did not show any antioxidant activity (Table 3) [83].

Furthermore, the antioxidant effect of *A. viridis* extract was investigated in Wistar rats with cyclophosphamide (CP)-induced endocrine dysfunction and reproductive toxicity. Rats received *A. viridis* extract (400, 200, and 100 mg/kg) for 30 days. The extract supplementation increased glutathione (GSH) levels and reduced thiobarbituric acid reactive substances (TBARS) in the brain and testis, respectively. The authors described that the therapeutic potential of *A. viridis* could be partly due to its antioxidant activity [84]. Rjeibi and coworkers [49] investigated the antioxidant effect of phytochemicals rich *A. spinosus* seed extracts on oxidative stress and liver toxicity induced by deltamethrin (DLM) in male Wistar rats. In the extract, TPC was 62.14 ± 2.3 mg GAE/g extract and TFC was 16.72 ± 1.02 mg catechin equivalent/g extract, as determined by reverse-phase high-performance liquid chromatography (RF-HPLC). The extract had a protective effect on the liver by decreasing MDA and enhancing activities of endogenous antioxidant enzymes including SOD, catalase (CAT), reduced glutathione (GSH), and glutathione peroxidase (GPx). Another in vivo study pretreated Wistar rats with *A. lividus* extracts for 9 days to determine the protective effects on CCl_4_-induced kidney damage. The phenolic compounds present in the aqueous extract of *A. lividus* ameliorated the oxidative stress in the kidney by increasing CAT activity. Moreover, the levels of MDA and myeloperoxidase (MPO) were significantly reduced. Overall, this study, for the first time reported the defensive effect of *A. lividus* extracts against CCl_4_-stimulated oxidative stress and kidney damage [85].

Balasubramanian et al. [86] observed the antioxidant effect of *A. hybridus* ethanol extract (AHELE) on liver and kidney of streptozotocin (STZ)-induced diabetic Wistar rats. The results showed that AHELE decreased the MDA and elevated the antioxidant status by increasing activities of CAT, SOD, and GSH in the liver and kidney. Biological activities may be attributed to the presence of saponins, flavonoids, tannins, and terpenoids in AHELE. Furthermore, the positive effects of *A. hypochondriacus* were demonstrated in hyperglycemic Wistar rats. The amaranth diet decreased total cholesterol and dipeptidyl peptidase IV activity in STZ-induced rats. Accumulation of apolipoprotein A-II and paraoxonase/arylesterase one (antioxidant) protein was observed in rats supplied with amaranth. The authors also suggested monitoring the level of triacylglycerol carefully, as the same was increased after amaranth supplementation. These findings indicate that amaranth has beneficial effects on reducing cholesterol (Table 3) [87].

### 3.4. Antioxidant Activity of Hydrolysates/Peptides from Amaranthus spp.

Concerning biofunctionality of hydrolyzed protein products, a number of studies have been published describing that amaranth protein hydrolysates/peptides could act as a natural antioxidant (Table 4). In an in vitro study, gastrointestinal (GI) digested *A. hypochondriacus* seeds sprout protein showed increased scavenging of 2,2’azinobis-(3-ethylbenzotiazoline-6-sulfonic acid (ABTS) radicals compared to its undigested counterpart. On the other hand, ORAC radicals’ scavenging was unchanged after in vitro digestion. The amaranth sprouts were suggested to have health-promoting ingredients [88]. The in vitro simulated direct GI digestion of the amaranth protein showed the IC_50_ values in electron spin resonance hydroxyl radical (ESR-OH), ORAC, and peroxynitrites (ONOO−) assay of peptides, were 25%, 20%, and 20%, respectively. However, the IC_50_ was found to be lower (50%) for the hydroxyl radical averting capacity (HORAC) assay. Notably, the radical scavenging activity of the digested protein with alcalase pretreatment failed to improve the antioxidant activity of *A. mantegazzianus* protein [89].

In continued research, Delgado et al. [90] screened GI digest for evaluating the antioxidant activity of peptide families of 11S globulin derived from *A. mantegazzianus*. Among nine peptide families, 10 peptides were assessed for their antioxidant activity, and four were found to be most active with their IC_50_ values ranging from 6.7 to 20 μg/mL. This is the first study to report the antioxidant activity of peptides from *Amaranthus* sp.

Recently, kiwicha (*A. caudatus*) protein concentrate (KPC) after GI digestion for 60 min (KD60) resulted in peptide fractions F1 and F2. The antioxidant activity against ORAC assay was found to be higher for F2 fraction than the F1 fraction. Overall, peptides (FLISCLL, SVFDEELS, and DFIILE) identified in fraction F2, displayed strong multipurpose activities such as antioxidant and capacity to inhibit α-amylase and angiotensin I-converting enzyme (ACE). Likewise, HVIKPPS showed antioxidant and inhibition of α-amylase activities in fraction F2 [91]. In another study, the radical scavenging activity of albumin 1 and globulin hydrolysates of *A. hypochondriacus* in ABTS and DPPH assay was approximately 40% [92].

Furthermore, Sabbione et al. [93] compared the radical scavenging activity of *A. hypochondriacus* protein isolate (I) and its hydrolyzed (by endogenous aspartic protease) product (H_EP_) in ABTS and ORAC assays. The results showed a substantial reduction in the IC_50_ values for the hydrolysate, suggesting more robust scavenging activity due to the existence of proteolysis-made bioactive peptides. The study also confirms the presence of an endogenous protease in *A. hypochondriacus*. In another study, for the first time, LAB, namely *Lactobacillus casei* (Shirota) and *Streptococcus thermophilus* (54102) were used for releasing bioactive peptides from raw amaranth isolated protein in mono and combined culture, and evaluated for DPPH, ABTS, and FRAP assays. In all assays, combined culture displayed the highest antioxidant activity [37]. This study highlights the importance of combined cultures over monocultures. Besides, the study shows symbiotic cooperation between amaranth protein and LAB. Furthermore, various LAB strains may also be used to identify the latest peptide-containing functional and biological activity.

Ayala-Niño et al. [94] screened protein hydrolysates (H1, H2, and H3) from seeds of *A. hypochondriacus* and evaluated DPPH, ABTS, and FRAP assays. The hydrolysate H3 exhibited the highest antioxidant activity on DPPH (388.9 μmol TE/100 g) and FRAP (592.5 μmol Fe^2+^/100 g) assays, while in ABTS assay, H1 hydrolysate showed the highest (425.8 mg TE/100 g) antioxidant activity. The H3 fraction was further fractionated using RP-HPLC, in particular, two fractions showed the highest scavenging of ABTS radicals with IC_50_ at 1.375 (fraction 22) and 0.992 (fraction 45) mg/L. Additionally, sequences were established using matrix-assisted laser desorption/ionization-time of flight and were found to be different from those acquired from common pepsin and pancreatin hydrolysates of amaranth proteins. The antioxidant peptides (from 0.5 to 1.4 kDa) obtained from fractions 22 and 45 were LVRW and DPKLTL, respectively, and their antioxidant activities were probably caused due to the presence of aromatic and hydrophobic amino acids. These findings indicate that alcalase and flavourzyme enzymes could contribute new bioactive peptides. Overall, the study concluded the presence of peptides with multifunctional activities, including antioxidant, ACE, and thrombin inhibitors.

In a recent study, the effect of storage and in vitro digestion of *Amaranthus* sp. microgreens on antioxidant capacity were evaluated. The storage categories include T1, T4, T7, and T10 based on the number of microgreens stored for 1, 4, 7, and 10 days, respectively. After 10 days of storage, DPPH and copper (II) reducing antioxidant capacity values increased significantly by 43% and 40%, respectively (Table 4). At the same time, for fresh microgreens, TAC showed reductions (18–20%) following 10 days of storage. Moreover, the phytoconstituents profile of microgreens changed during storage conditions. This study highlights the importance of storage time and storage conditions on function attributes of microgreens following digestion [95]. Another study evaluated the antioxidant capacity using one-stage and two-stage (combination) enzymatic treatments using food-grade commercial enzymes and in vitro simulated digestion. The highest (72.8%) antioxidant activity was shown by alcalase-neutrase combination with the lowest IC_50_ value of 0.29 mg/mL. Nevertheless, in vitro simulated digestion showed no significant effect on the antioxidant activity [96]. In a different study, protein hydrolysates (alcalase, chymotrypsin, pepsin, and trypsin) and the ultra-filtered peptide fractions of the pepsin hydrolysate of amaranth protein were evaluated for antioxidant activity. The peptide fractions ranged from <1 to <10 kDa. The radical scavenging capacity of pepsin hydrolysate was found to be higher than the other protein hydrolysates. However, when compared with peptide fractions, pepsin hydrolysate showed weak metal chelating activities.

In addition, the lower molecular weight (<1 kDa) fraction demonstrated better scavenging in tested assays compared with the higher molecular weight (>1 kDa) fractions [97]. In another study, the antioxidant potential of alcalase, pepsin, and trypsin hydrolysates of *A. cruentus* protein was found to be higher than unhydrolyzed protein. In DPPH assay, the highest (34.41 μg/mL) and lowest (23.06 μg/mL) IC_50_ values were reported for trypsin and pepsin hydrolysate, respectively. The IC_50_ values in FRAP assay of alcalase, trypsin, and pepsin hydrolysates were 23.92, 25.29, and 28.28 μg/mL, respectively. In ABTS assay, the highest and lowest IC_50_ values were reported for alcalase hydrolysate (206.6 μg/mL) and trypsin hydrolysate (114 μg/mL), respectively [98]. The antioxidant activity of germinated amaranth flour (GAF) was increased by 54.3% compared to ungerminated amaranth flour (UAF). The ORAC values for the hydrolyzed GAF and UAF were 1304.9 and 983.1 μmol TE/mg soluble protein, respectively [99]. These findings suggest that the combined action of germination and enzymatic hydrolysis could increase the antioxidant capacity of the amaranth plant. Nevertheless, not many in vivo experiments have been carried out hitherto, so the precise effect on the human body is not understood.

## 4. Conclusions and Prospects

*Amaranthus* spp. is an underutilized and ignored plant despite its high protein and nutrient content. The plant offers excellent antioxidant activity compared to cereals. A special feature of amaranth is its hydrolysates and bioactive peptides with superior antioxidant activity compared to the parent isolate. The review offers analysis on recent in vitro and in vivo work supporting the excellent free radical scavenging activity of amaranth. Indubitably, it appears that phytoconstituents of amaranth might play an important role in direct scavenging of free radicals, activation of enzymatic and nonenzymatic antioxidants, as well as the Keap1/Nrf2/ARE signaling pathway.

However, the latest studies underline the beneficial impact of hydrolysates and bioactive peptides of *Amaranthus* spp. as free radical scavengers and mainly influenced by peptide molecular weight, structure, and its amino acid composition. Recently, the use of LAB fermentation to release bioactive peptides from amaranth protein has been applied and progress in this field depends mainly on the discovery of novel strains for the generation of higher efficiency bioactive peptides. Additionally, sequential enzymatic hydrolysis and identification of amaranth peptide have also contributed to the production of novel bioactive peptides with multiple biological roles, including antioxidant, ACE, and thrombin inhibitors. In light of this, future studies should concentrate on identifying bioactive peptides from *Amaranthus* spp. with multivariate applications. In addition, in vivo and ex vivo experimental validation to test their actual potential as bioactive molecules of pharmaceutical importance should be carried out. Safety of peptides, however, in commercial products is a must. Furthermore, computational approaches could be used for initial screening and prediction of potential precursors of bioactive peptides. Hydrolysates and bioactive peptides derived from *Amaranthus* spp. could begin to grow as a substitute for synthetic antioxidants in nutraceutical and functional food industries.

Furthermore, phytoconstituents, macronutrients, minerals, and dietary fiber are fortunate to be higher in *Amaranthus* spp. than most traditional cereals; therefore, their utilization in gastronomy is very promising. In light of these issues, one remedy is to develop tasty and nutritious recipes that satisfy the requirements of the modern kitchen. However, the main challenge is to standardize such recipes by using gastronomy practices that retain essential nutritional elements. Besides, the growth of the amaranth market is relatively slow, in part because there is a general lack of experience in the private–public sectors. Awareness and accessibility of amaranth should be improved to tackle this issue. Overall, expanding research on *Amaranthus* spp. shows the plant can manage and prevent chronic ailments related to oxidative stress. The awareness of this plant is getting bigger and this is why it is worth updating the subject regularly.

## Figures and Tables

**Table 1:** Antioxidant and radical scavenging activity of *Amaranthus* spp.

Species	Material	Country	Extract	Methods/Findings	Ref.
*A. spinosus*	Leaves	Ethiopia	80% methanol	DPPH IC_50_ = 83.5 μg/mL; LAS = 67.4%	[38]
Organic Amaranth	Gluten-free flour	USA	Free-ethanol Bound-2 N NaOH	DPPH = Free: 0.13–0.97 μmol/g; Bound: 9.8–9.9 μmol/g	[39]
Tannin from *A. caudatus*	Leaves, flowers, and seeds	Korea	Methanol and hot water	DPPH (RC_50_) = PFM: 155.1 μg/mL, RLM: 189.9 μg/mL, (GLM, SHW, SM, RFM, GLHW, PFHW, RFHW, RLHW) ≥1000 μg/mL; ABTS (RC_50_) = PFM: 195.6 μg/mL, RLM: 166.9 μg/mL, RLHW: 303.3 μg/mL, PFHW: 321.7 μg/mL, (GLM, SM, RFM, GLHW, RFHW, SHW) ≥1000 μg/mL	[40]
*A. hypochondriacus*, *A. caudatus*, and *A. cruentus*	Flower, leaf, seed, sprout, and stalk	Canada	80% methanol	FRAP = 0.6–62.2 μmol AAE/g DW; ORAC = 30.7–451.4 μmol TE/g DW	[41]
*A. lividus* and *A. tricolor*	Leaves	Thailand	Petroleum ether, methanol, and dichloromethane	DPPH = 1.7 ± 0.9–15.2 ± 1.6 mg VCEAC/g dry plant material ABTS = 2.1 ± 0.9–25 ± 1.6 mg VCEAC/g dry plant material	[42]
*A. caudatus, A. viridis,* and *A. lividus*	Leaves	Sri Lanka	Methanol	DPPH = 4.3 ± 3.2–24.3 ± 1.6%; RP = 0.7 ± 0.1–4.4 ± 0.1 mg AAE/g DW leaf; LPO = 79.2 ± 1.8–84.5 ± 6.3; TAC = 5.7 ± 0.3–15.8 ± 0.46 mg AAE/g DW	[43]
*A. lividus* and *A. hybridus*	Stem and seeds	Bangladesh	Methanol	DPPH IC_50_ = *A. lividus* (93 ± 2.4 μg/mL) and *A. hybridus* (28 ± 1.8 μg/mL)	[44]
*A. caudatus*	Germinated seeds	Canada	90% methanol	DPPH = 43.2 ± 1.1–110.7 ± 1.3 mg Trolox/100 g db; ABTS = 52.3 ± 2.1–129.8 ± 4.5 mg Trolox/100 g db	[45]
*A. caudatus*	Germinated seeds	Peru	Methanol	DPPH = 149.6 ± 4.5–151.8 ± 5.1 mg TE/g of sample; ABTS = 155.9 ± 10–180.2 ± 8 mg TE/g of sample; FRAP = 102.4 ± 6–136.4 ± 8 mg TE/g of sample	[46]
*A. viridis*	Leaves	Nigeria	Ethanol	DPPH IC_50_ = 107.8%; NO IC_50_ = 72.2%; LPO IC_50_ = 78.1%; FTC IC_50_ = 78.1%	[47]
*A. viridis*	Leaf, seed, and stem	Malaysia	Methanol	DPPH IC_50_ = leaf, 115.7 ± 1.6 μg/mL, seed, 189.2 ± 1.3 μg/mL, stem, >300 μg/mL; NO IC_50_ = leaf, 244.36 ± 2.15 μg/mL, seed, 299.4 ± 1.3 μg/mL, stem, >300 μg/mL; FRAP IC_50_ = leaf, 63.5 ± 1.8 μg/mL, seed, 83.5 ± 1.3 μg/mL, stem, >300 μg/mL	[48]
*A. spinosus*	Seeds	Tunisia	Methanol	DPPH = 84.1%; H_2_O_2_ = 111.1 ± 8.4 μg/mL	[49]
*A. caudatus*, *A. cruentus*, *A. hybrid*, *A. hypochondriacus A. hybridus*	Seeds	USA and Nigeria	Methanol	DPPH = 89.5 ± 0.5–93.4 ± 1.3 g/kg; ABTS = 169.6 ± 3.8–201.5 ± 4.1 mmol TE/100 g; TAC = 140.2 ± 4.9–199.9 ± 16.5 mg AAE/100 g; FR = 0.14–0.19 g/100 g; FC = 57.5 ± 3.3–66.7 ± 6.4 g/kg	[50]
*A. acanthochiton, A. blitum, A. caudatus, A. cruentus, A. deflexus, A. dubius, A. graecizans, A. hybr., A. hybridus, A. palmeri, A. retroflexus, A. spinosus, A. tricolor, A. virindis,* and *A. thunbergii*	Leaves	USA	50% methanol	ORAC = 38 ± 8.1–90 ± 7.3 mmol TE/g	[51]
*A. cruentus, A. hybridus, A. caudatus, A. hypochondriacus, X hybridus, A. hypochondriacus*	Leaves	Italy	Methanol	DPPH IC_50_ = 1.7–3.4 mg/mL; ABTS IC_50_ = 0.97–2.2 mg/mL	[52]
*A. caudatus*	Sprouts	Peru	Methanol:HCl:water (80:0.1:19.9)	ORAC = 369.5–2525.6 mg TE/100 g DW	[53]
*A. spinosus*	Leaves	Tunisia	80% methanol	DPPH EC_50_ = 25.6–54.6 μg/mL; H_2_O_2_ EC_50_ = 37.5–116.7 μg/mL	[54]
*A. tricolor*	Leaves	India	Chloroform, methanol, and water	DPPH IC_50_ = 290 (methanol), 657 (chloroform), 830 (water) μg/mL; p-NDA IC_50_ ≥1000 μg/mL for methanol, chloroform, water	[55]
*A. cruentus*	Stem	Korea	70% ethanol	DPPH IC_50_ = 279.9–1041.4 μg/mL; ABTS IC_50_ = 798.4–2672.6 μg/mL	[56]
*A. spinosus*	Leaves	Tunisia	80% ethanol, *n*-hexane, water, *n*-butanol, ethyl acetate, and chloroform	TAC EC_50_ = 45.5 ± 0.3–90.0 ± 2.1 μg/mL; DPPH EC_50_ = 27.3 ± 0.8–90.7 ± 5.2 μg/mL; H_2_O_2_ EC_50_ = 30.6 ± 1.2–258.9 ± 8.5 μg/mL	[57]
Vegetable amaranth genotypes (VA6, VA11, VA14, and VA16)	Leaves	Bangladesh	90% methanol	DPPH = 2.3 ± 0.1–37.5 ± 0.2 TEAC μg/g DW; ABTS = 26.7 ± 0.3–81.8 ± 0.2 TEAC μg/g DW	[58]
VA6, VA11, VA14, and VA16	Leaves	Bangladesh	90% methanol	Significant increase in TAC Highest: VA16, Lowest: VA11 LDS < MDS < SDS	[59]
*A. tricolor*	Leaves	Bangladesh	90% methanol	DPPH = 23.5 ± 0.3–44.8 ± 0.2 TEAC μg/kg DW; ABTS = 52.4 ± 0.6–82.5 ± 0.8 TEAC μg/kg DW	[60]
Vegetable amaranth	Leaves	Bangladesh	90% methanol	DPPH = 14.99–32.83 TEAC μg/g DW	[61]
*A. hybridus*	Polysaccharides AHP-M-1, AHP-M-2	China		DPPH = AHP-M-1: 72.4%, AHP-M-2: 78.9%; H_2_O_2_ = AHP-M-1: 32.6%, AHP-M-2: 40.2%; superoxide ion = AHP-M-1: 63.9%, AHP-M-2: 80.2%; Fe^3+^–Fe^2+^ reduction = AHP-M-1: 0.57, AHP-M-2: 0.90	[62]
*A. hybridus*	Polysaccharides AHP-H-1, AHP-H-2	China		DPPH = AHP-H-1: ~80%, AHP-H-2: >80%; H_2_O_2_ = AHP-H-1: 40%, AHP-H-2: 48%; superoxide ion = AHP-H-1: 80%, AHP-H-2: >60%; Fe^3+^–Fe^2+^ = AHP-H-1: 0.695, AHP-H-2: 0.918	[63]
*A. viridis* (WAV4, 7, and 9), *A. spinosus* (WAS11, 13, and 15)	Leaves	Bangladesh	90% methanol	DPPH = WAV: 21.9 ± 0.1–24.7 ± 0.1 TEAC μg/g DW, WAS: 25.9 ± 0.1–27.6 ± 0.1 TEAC μg/g DW; ABTS = WAV: 45.8 ± 0.4–51.2 ± 0.2 TEAC μg/g DW, WAS: 47.9 ± 0.3–52.4 ± 0.2 TEAC μg/g DW	[64]
Red amaranth (RA1 to RA25)	Leaves	Bangladesh	90% methanol	DPPH = RA1: 11.2–RA25: 31.7 TEAC μg/g DW; ABTS = RA1: 21.8–RA25: 62 TEAC μg/g DW	[65]
*A. tricolor* and *A. lividus*	Leaves	Bangladesh	90% methanol	DPPH = 43.8 TEAC μg/g DW; ABTS = 66.6 TEAC μg/g DW	[66]
*A. lividus*	Leaves	Bangladesh	90% methanol	DPPH = 9.21 ± 0.1–26.6 ± 0.2 TEAC μg/g DW; ABTS = 16.7 ± 0.1–51.7 ± 0.03 TEAC μg/g DW	[67]
Green morph amaranth	Leaves	Bangladesh	90% methanol	DPPH = 8.90 ± 0.14–26.56 ± 0.13 TEAC μg/g DW; ABTS = 16.84 ± 0.33–48.12 ± 0.13 TEAC μg/g DW	[68]
*A. blitum*	Leaves	Bangladesh	90% methanol	DPPH = 12.3 ± 0.1–29.5 ± 0.2 TEAC μg/g DW; ABTS = 21.9 ± 0.1–55.7 ± 0.03 TEAC μg/g DW	[69]
Amaranth grain variety (VL-44)	Cookies from raw and germinated seeds	India		DPPH = raw: 15.1 g/100 g; germinated: 21.4 g/100 g	[70]
Amaranth grain variety (K432)	Grain	India	Water/methanol (50:50) and acetone/water (70:30)	DPPH = 16.4 g GAE/kg; ABTS = 15.3 g GAE/kg	[71]
*A. spinosus*, *A. dubius*, *A. viridis*, and *A. tricolor*	Aerial portions	India	100% methanol	DPPH = 63.94 ± 3.72–92.20 ± 4.21 µg/mL; H_2_O_2_ = 26.02 ± 1.50–32.13 ± 1.36 µg/mL; Ferric reducing = 20.44 ± 0.94–30.04 ± 1.20 µg/mL	[72]

DPPH-1,1-diphenyl-2-picrylhydrazyl; LAS, linoleic acid system; PFM, purple flower extracted with methanol; RLM, red leaves extracted with methanol; GLM, green leaves extracted with methanol; SHW, seeds extracted with hot water; SM, seeds extracted with methanol; RFM, red flower extracted with methanol; GLHW, green leaves extracted with hot water; PFHW, purple flower extracted with hot water; RFHW, red flower extracted with hot water; RLHW, red leaves extracted with hot water; ABTS, 2,2’-azino-bis(3-ethylbenzothiazoline-6-sulfonic acid); I,R,EC_50_, 50% reduction concentration; FRAP, ferric reducing antioxidant power; ORAC, oxygen radical absorbance capacity; AAE, ascorbic acid equivalent; TE, Trolox equivalent; DW, dry weight; VCEAC, vitamin C equivalent antioxidant capacity; RP, reducing power; LPO, lipid peroxidation, TAC, total antioxidant capacity; db, dry basis; NO, nitric oxide; FTC, ferric thiocyanate; FR, ferric reducing; FC, ferric chelating; p-NDA, p-Nitroso dimethyl aniline; LDS, low drought stress; MDS, medium drought stress; SDS, severe drought stress; TEAC, trolox equivalent antioxidant capacity; GAE, gallic acid equivalents.

**Table 2:** In vitro studies showing the antioxidant capacity of *Amaranthus* spp.

Species	Country	Material/Solvent	Cell Line	Dose	Major Findings	Ref.
*A. viridis*	India	Alkaloids from leaves/methanol	H_2_O_2_-induced human erythrocytes	25, 50 mg	A dose-dependent increase in the SOD, CAT, GST, GSH, and VC	[81]
*A. lividus* and *A. tricolor*	Thailand	Leaves, petroleum ether, dichloromethane, and methanol	H_2_O_2_-treated human neuroblastoma (SH-SY5Y) cell line	1.56, 3.07, 6.25, 12.5, 25, 50, 100 μg/mL	Significant reduction of ROS dose. *A. tricolor* all extracts (Effective dose—100 μg/mL) *A. lividus*-petroleum ether and the methanol (effective dose—100 μg/mL), dichloromethane—50 μg/mL)	[42]
*A. cruentus*	Germany	Leaves/ethanol	AFB1 and oxidative stress-induced HepG2	1.4, 4.1, 12.3, 37.0, 111.1, 333.3 μg/mL	Decreased ROS production in a concentration-dependent manner. Induction of ARE/Nrf2-mediated antioxidant enzymes	[82]

H_2_O_2_, hydrogen peroxide; SOD, superoxide dismutase; CAT, catalase; GST, glutathione-S-transferase; GSH, glutathione; VC, vitamin C; ROS, reactive oxygen species, AFB1, Aflatoxin-B1; ARE, antioxidant responsive element; Nrf2, nuclear factor erythroid 2-related factor 2.

**Table 3:** In vivo studies showing the antioxidant capacity of *Amaranthus* spp.

Species	Country	Material	Model	Treatment	Methods/Findings	Ref.
*A. mantegazzianus* protein	Argentina	Seed flour	Wistar rats	C, Chol (chol 1%, *w/w*), CE (E 0.005%, *w/w*), CholE (chol 1%+E 0.005%, *w/w*), CAI (AI 2.5%, *w/w*), CholAI (chol 1%+AI 2.5%, *w/w*)	↑ antioxidant capacity of the plasma by CholAI ↓ lipid oxidation products level of plasma and liver	[83]
*A. viridis*	Nigeria	Leaves	CP-induced Wistar rats	CP+100, 200, and 400 mg/kg of *A. viridis*	↑ GSH levels in the brain and testis ↓ TBARS activity in the brain and testis	[84]
*A. spinosus*	Tunisia	Seeds	DLM-induced Wistar rats	250 mg/kg BW for 1 h	↓MDA levels ↑ SOD and CAT activities and GPx, GSH levels	[49]
*A. lividus*	Turkey	Stem with leaves and flowers	CCl_4_-induced Wistar rats	Pretreatment with *A. lividus* 250 and 500 mg/kg BW for 9 days and 10th day-CCl_4_ (1.5 mL/kg BW; 50% in olive oil)	*A. lividus* supplementation prevented the decrease of CAT activity No significant effect on GST, GPx, GR, and SOD activities	[85]
*A. hybridus*	India	Leaves	STZ induced Wistar albino rats	AHELE at a dose of 200 and 400 mg/kg, for 14 days	Dose-dependent ↑ of SOD and CAT activities and GSH levels in the liver and kidney and ↓ MDA levels	[86]
*A. hypochondriacus*	Mexico	20% popped amaranth grain	STZ induced Wistar rats	30 g, 12 weeks	↓DPP-IV activity and TC Accumulation of Apo-A-II and PON1 proteins	[87]

C, basic diet; Chol, cholesterol; E, α-tocopherol; AI, protein isolate from *A. mantegazzianus*; CP, cyclophosphamide; GSH, glutathione; TBARS, thiobarbituric acid reactive substances; DLM, deltamethrin; MDA, malondialdehyde; SOD, superoxide dismutase; CAT, catalase; GSH, reduced glutathione; GPx, glutathione peroxidase; CCl_4_, carbon tetrachloride; GST, glutathione-S-transferase; GR, glutathione reductase; STZ, streptozotocin; AHELE, *A. hybridus* ethanol leaf extract; DPP-IV, dipeptidyl peptidase IV; TC, total cholesterol; Apo-A-II, apolipoprotein A; PON1, paraoxonase/arylesterase 1.

**Table 4:** Antioxidant activity of hydrolysates and peptides derived from various *Amaranthus* spp.

Species	Country	Enzyme/Bacteria	Peptides/Hydrolysates	Peptide Sequence	Findings	Ref.
*A. hypochondriacus*	Argentina	Pepsin and pancreatin	Hydrolysate		ABTS = Seed sprout isolate (SI) undigested (0.32) SI digested (0.72) μmol/mg	[88]
*A. mantegazzianus*	Argentina	Pepsin and pancreatin	Hydrolysate	-	IC_50_ = ESR-OH (25%), ORAC (20%), peroxynitrites (20%), and HORAC (50%)	[89]
*A. mantegazzianus*	Argentina	Pepsin and pancreatin	Hydrolysate and peptides	AWEEREQGSR (1) YLAGKPQQEH (2) IYIEQGNGITGM (3) TEVWDSNEQ (4)	IC_50_ ORAC = (1): 6.7 μg/mL, (2): 16 μg/mL, (3): 17μg/mL, (4): 20 μg/mL	[90]
*A. caudatus*	Ecuador	Pepsin and pancreatin	Hydrolysate and peptides	F1 (YESGSQ, GGEDE, NRPET), F2 (FLISCLL, TALEPT, HVIKPPS, SVFDEELS, ASANEPDEN, DFIILE)	ORAC values = F2 fraction 4.47 μmol TE/mg peptide; F1 fraction 1.56 μmol TE/mg peptide	[91]
*A. hypochondriacus*	USA	Alcalase	Hydrolysate	-	DPPH and ABTS (40%)	[92]
*A. hypochondriacus*	Argentina	Endogenous protease	Hydrolysate (H_EP_)	-	ABTS; Protein isolate (I) = 5.40 ± 0.50 mg/mL; H_EP_ = 2.1 ± 0.3 mg/mL. ORAC; I = 0.102 ± 0.021 mg/mL; H_EP_ = 0.058 ± 0.027 mg/mL	[93]
Raw *amaranth* seeds	Mexico	LAB	Hydrolysate	-	DPPH = 104.1 ± 9.7–168.1 ± 5.7 μmol TE/mL; ABTS = 103.9 ± 10–268.4 ± 11.8 μmol TE/mL; FRAP = 225.6 ± 19.6–381.3 ± 0.6 μmol Fe^2+^/mL	[37]
*A. hypochondriacus*	Mexico	Alcalase and flavourzyme	Hydrolysate H1, H2, and H3	LVRW, DPKLTL	DPPH = 76.6 ± 1.6–388.9 ± 2.7 μmol TE/100 g; ABTS = 115.6 ± 10.3–425.8 ± 0.6 mg TE/100 g; FRAP = 63.3 ± 5.7–592.5 ± 29.2 μmol Fe^2+^/100 g	[94]
*Amaranthus* spp.	Turkey	Pepsin and pancreatin	Hydrolysate	-	DPPH = T1 (379.5 ± 14.6), T4 (309.4 ± 36.9), T7 (423.8 ± 31.7), T10 (542.8 ± 33.6) mg TE/100 g DW; CUPRAC = T1 (825.7 ± 9.0), T4 (628.0 ± 8.2), T7 (947.7 ± 9.6), T10 (1158.5 ± 12.2) mg TE/100 g DW	[95]
*A. caudatus*	Peru	Alcalase, neutrase, flavourzyme, and their combination	Hydrolysate	-	IC_50_ ABTS = 0.29 mg/mL	[96]
*Amaranthus* sp.	Nigeria	Alcalase, trypsin, pepsin, and chymotrypsin	Hydrolysate	-	EC_50_ (mg/mL) = DPPH (0.35 ± 0.01–1.09 ± 0.07); superoxide (0.91 ± 0.03–0.98 ± 0.10); hydroxyl (0.35 ± 0.04–1.09 ± 0.07); metal chelation (3.76 ± 0.08–4.81 ± 0.09)	[97]
*A. cruentus*	South Africa	Alcalase, trypsin, and pepsin	Hydrolysate	-	IC_50_ (μg/mL) = DPPH (23.06–34.41); FRAP (23.92–28.28); ABTS (114–206.6)	[98]
*A. hypochondriacus*	Mexico	Pepsin and pancreatin	GAF, UAF hydrolysate	-	After enzymatic hydrolysis (at 270 min) ORAC UAF (983.1) and GAF (1304.9) μmol TE/mg soluble protein	[99]

ABTS, 2, 2’-azinobis (3-ethylbenzothiazoline-6-sulfonic acid); ESR-OH, electron spin resonance hydroxyl radical; ORAC, oxygen radical absorbance capacity; HORAC, hydroxyl radical averting capacity; IC_50_–50% reduction concentration; DPPH,1,1-diphenyl-2-picrylhydrazyl; LAB, lactic acid bacteria; FRAP, ferric reducing antioxidant power; TE/mL, Trolox equivalents per milliliter; DW, dry weight; CUPRAC, copper (II) reducing antioxidant capacity; GAF, germinated amaranth flours; UAF, ungerminated amaranth flour.
